# The effect of ultrasound on birch sawdust during simultaneous pretreatment and hemicellulose’s chemical conversion^☆^

**DOI:** 10.1016/j.ultsonch.2025.107318

**Published:** 2025-03-19

**Authors:** Salla Kälkäjä, Tao Hu, Stéphane Baup, Jean-Marc Lévêque, Katja Lappalainen

**Affiliations:** a Sustainable Chemistry Research Unit, University of Oulu, P.O. Box 4300, FIN-90014 Oulu, Finland; b Université Grenoble Alpes, CNRS, Grenoble INP, LRP, 38000 Grenoble, France

**Keywords:** Birch sawdust, Furfural, Lignocellulosic side stream, Ultrasound assisted hydrolysis

## Abstract

The effect of low-frequency ultrasound on the treatment of birch sawdust with hot water and diluted acid was studied for the first time under high pressure and temperature. When treated with diluted formic acid solution, the production of furfural dramatically increased, emphasizing the straightforward hydrolysis of hemicellulose. The highest furfural yield (43–44 %) was achieved with 5 % formic acid. The most significant decrease in crystallinity index (from 56 % to 50 %) compared to treatment without ultrasound was observed with 2.5 % formic acid, using a 70:30 pulse mode with 70 % amplitude of the nominal power deliverable by the generator and an additional pressure of 3 bar. In diluted acid experiments, the remaining solid fraction, which presumably contained mainly cellulose and lignin, exhibited a completely different shape compared to the starting material, resulting in a kind of “wooden paste” with reduced particle size. The impact of additional pressure appeared to be significant, and this is further discussed in relation to the determination of the acoustic power under such rigorous experimental conditions. Based on the results obtained, ultrasound combined with hot water / diluted acid treatment shows potential for the efficient utilization of birch sawdust for platform chemicals.

## Highlights

• Birch sawdust was pretreated at high pressure and temperature using ultrasound.
• Ultrasound combined with diluted acid resulted in unstructured wood and furfural.
• Ultrasound reduced the crystallinity of birch sawdust.
• Cellulose pretreatment and hemicellulose conversion to furfural were achieved.

## Graphical abstract

**Figure:**
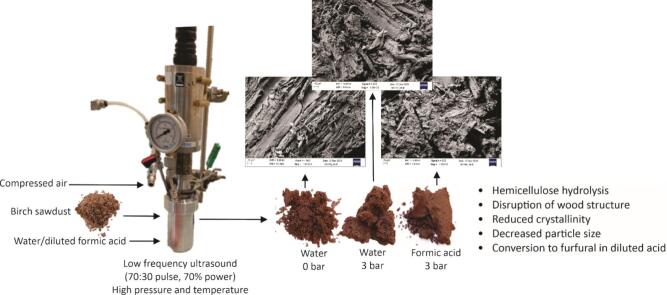

## 1 Introduction

Increasing the economic competitiveness and sustainability of biorefining compared to oil refinery is of crucial importance to promote the transition from petroleum-based solutions to a large-scale sustainable bioeconomy. The competitiveness of biorefining can be increased, for example, by improving overall processes, using renewable energy, and increasing feedstock utilization efficiency. Utilizing side streams to create various bio-based value-added products is one major aspect in the overall utilization efficiency of biomass [1,2]. When considering possible side streams, it is important to note that in Europe, most bio-refineries are associated with the pulp, paper, timber, and chemical industries [3]. Therefore, wood-derived side streams are constantly available. However, the composition of wood varies among species [4], which impacts the potential utilization purposes of side streams.

The sawdust of hardwoods, such as birch, is a versatile raw material for platform chemical production. It is mainly composed of lignin, cellulose, and hemicellulose. The versatility of birch sawdust is due to the composition of hemicellulose. Birch hemicellulose is composed mainly of xylan polymer, which makes the hemicellulose rich in pentose units [5,6]. Therefore, by separating the main components (hemicellulose, cellulose, and lignin) into individual fractions, they can be processed to produce different platform chemicals. Whereas hemicellulose fraction can be used for furfural production, cellulose fraction can be converted into 5-hydroxymethyl furfural (HMF), and lignin can be processed further to obtain different aromatic compounds or used for the development of new heterogeneous catalysts [[7], [8], [9]]. In addition to its high C5 sugar content, birch hemicellulose is partially acetylated [10], and the acetyl groups can be cleaved off during hemicellulose hydrolysis. The release of bound acetyl groups has been shown to increase with increasing severity of the treatment [11]. Released acetic acid increases the overall acidity of the medium and promotes catalytic hydrolysis in conversion reactions [12]. This contributes to one of the major concepts of green chemistry by decreasing the necessary amount of added chemicals to perform a chemical transformation [13].

Different chemical, biological, physical/mechanical methods, or combination of those can be used for biomass fractionation [14]. Ultrasound is considered a green technology because it is linked to shorter treatment times, lower operating temperatures, and overall increase of treatment efficiency in combined methods, usually with less added chemicals [15,16]. Interestingly, the literature also reveals that ultrasound is able to improve the selectivity of some conversion reactions [17].

Ultrasound is based on acoustic waves at frequencies above human hearing, from 18 kHz up to a hundred of MHz, but only a small window allows for physical/chemical effects on matter, namely 20–2000 kHz. In this frequency range, two broad sub-windows can be defined, with different effects on matter, that is, either chemical or physical. However, whatever the applied frequencies, direct and indirect effects of ultrasound on matter arise from the unique phenomenon of cavitation, which is the birth, growth, and collapse of micrometric bubbles of gas into the irradiated liquid [16,18]. At low frequencies (20–100 kHz), the collapse of the cavitation bubbles is rather low and promotes mechanical effects (e.g., acoustic streaming, heat, and mass transfer intensification). At higher frequencies (>200 kHz), bubbles collapse violently, as described by hot-spot theory, involving very high temperature and pressure conditions and the production of transient radical species, leading to chemical transformations [16,19,20].

According to the literature, increasing temperature decreases the sonochemical effect. However, increasing ambient pressure has been observed to increase the sonochemical effects overall. It has been suggested that this kind of behavior is due to changes in vapor pressure. When the temperature increases, the equilibrium vapor pressure of the system also increases, and increasing vapor pressure causes a cushioning effect because the formed cavitation bubbles contain more solvent vapor, even though generally bubble formation is easier under higher temperatures. On the other hand, increasing pressure intensifies cavitation by decreasing the vapor pressure and the formed bubbles contain less solvent vapor [21]. However, since hydrolysis reactions of both hemicellulose and cellulose require high temperatures [[22], [23], [24]], it can be expected that additional pressure may counterbalance the potential cushioning effect of high temperature in a closed vessel.

Typically, ultrasound has been used as an efficient pretreatment method for biomass or as an intensification of other methods [25]. According to the literature, ultrasound has been applied mainly in atmospheric pressure which sets limitations for temperature. Only a few studies were found where ultrasound was applied under high pressure to obtain plant polysaccharides, but in those studies, the temperature was kept under 100 °C [[26], [27], [28]]. To the best of our knowledge, studies using high pressure and temperature conditions have not been published before. Therefore, the effect of ultrasound under pressure and high temperature needs to be studied further.

Even if conditions similar to those used in this study have not been studied previously, it has been shown that ultrasound has various effects on lignocellulose components. It can reduce the length and diameter of cellulose fibers, damage the fiber surface, and decrease the crystallinity [29]. All the mentioned factors promote easier cellulose degradation. Sivakumar et al. demonstrated that sonication combined with acid hydrolysis increased the saccharification rate compared to silent acid hydrolysis [30]. The authors studied the effect of ultrasound on eucalyptus’ physicochemical structure and noted that ultrasound treatment reduced the amount of alkali metals in solid fraction and increased crystallinity. However, the increase of crystallinity was most likely due to the degradation of amorphous hemicellulose/lignin. Ultrasound also promoted the accessibility of the liquid medium by disrupting the lignocellulosic structure and creating microchannels [31]. Ultrasound pretreatment has also been observed to enhance the extraction efficiency of hemicellulose and phenolic compounds from bamboo bast fiber powder in hot water [32]. Despite the previously reported results of ultrasound’s ability to decrease cellulose crystallinity and disrupt the lignocellulosic structure, it has also been found that applying ultrasound can enhance the condensation reaction of lignin and in fact cause repolymerization of smaller units [33,34]. Therefore, the ultrasound parameters must be evaluated and optimized so that the desired effects are maximized.

**Fig. 1:**
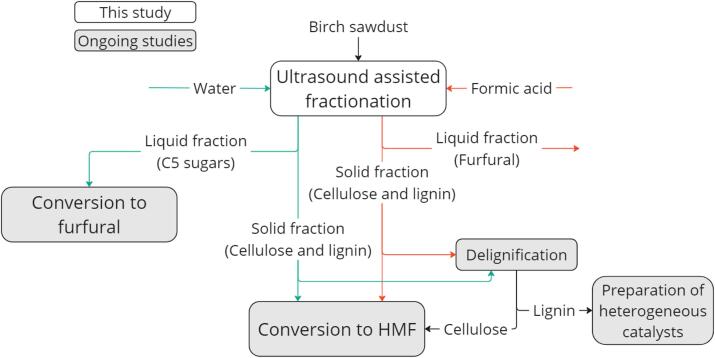
Diagram of conducted and ongoing studies on the ultrasound-assisted fractionation of birch sawdust and conversion to platform chemicals.

The aim of this research was to use ultrasound assisted method for simultaneous pretreatment and hemicellulose hydrolysis/conversion (Fig. 1). The aims for the pretreatment were decreasing particle size, disrupting the wood structure and lowering cellulose crystallinity. According to the literature these factors enable easier cellulose hydrolysis which facilitates the HMF production in further steps [8,35,36]. Even if ultrasound is generally related to lower operating temperatures, it must be emphasized that in this study the operating temperature was intentionally increased, since to date the effect of ultrasound on the biomass side stream at such harsh conditions has not been explored. The two reasons for using harsh conditions are that it is interesting to explore the behavior and impact of low-frequency ultrasonic waves at such high temperature and pressure to increase overall knowledge about this technology, as well as that hemicellulose and cellulose undergo hydrolysis at high temperature, respectively at 180–200 °C [23,37] and 250–280 °C [22,38].

## 2 Materials and methods

### 2.1 Chemicals

The birch sawdust was obtained from a sawmill in northern Sweden, and it was dried at 55 ˚C before use. The composition analysis of birch sawdust has been described by Kälkäjä et al. as consisting of 3.1 % extractives, 0.6 % ash, 27.4 % lignin, 33.9 % cellulose, and 29.8 % hemicellulose [39]. The hemicellulose composition (67 % xylose, 10 % glucose, 4 % mannose, 4 % galactose, 3 % arabinose, and 2 % rhamnose of the dry basis) has been determined in a previous study by Lempiäinen et al. [40]. Deionized water was used in all the experiments and solutions. Ultrapure water was used for the liquid chromatography. Formic acid (Merck, ≥98 %), methanol (Sigma Aldrich, ≥99.9 %), trifluoroacetic acid (TFA; Honeywell, ≥99.0 %), furfural (Sigma Aldrich, 99 %), 5-hydroxymethyl furfural (HMF; Acros Organics, 98 %), acetonitrile (ACN; ≥99.9 % Fischer Scientific), triethylamine (TEA; >99 %, TCI), D-(+)-xylose (99 %, Acros Organics), D-(+)-glucose anhydrous (≥99.5 %, Fluka BioChemika), and deuterium oxide (D_2_O; 99.9 %, VWR Chemicals) were used as received without further purification.

### 2.2 Ultrasound experiments

#### 2.2.1 Acoustic power measurement

**Fig. 2:**
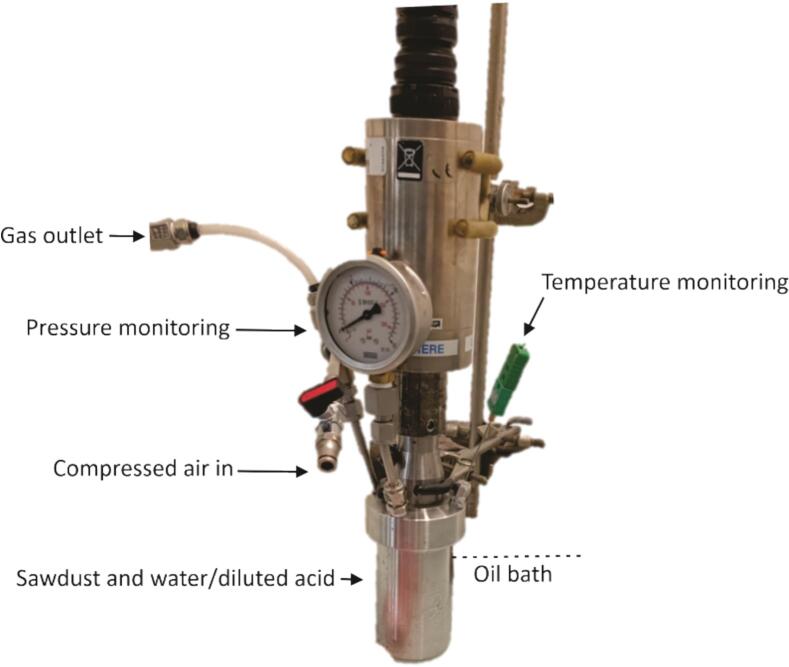
Experimental setup for ultrasound experiments.

The acoustic power measurements were obtained using a Branson sonifier 450 (20 kHz) fitted to a stainless-steel batch reactor equipped with a manometer, a temperature sensor, and a gas inlet/outlet system, facilitating the adjustment of the internal pressure. The experimental setup is illustrated in Fig. 2.

The acoustic power was determined by a classical calorimetric method (equation (1) during a certain lapse of time [41] at two different starting temperatures, 22 °C and 150 °C (Fig. 2). For each starting temperature, the initial pressure was either equal or three times the atmospheric pressure created by compressed air to measure the impact of added pressure on both thermic systems. In the measurements conducted at 150 °C, the pressure was 6.5 and 3 bar in the beginning of experiment with and without additional pressure, respectively. In addition, two pulse modes of the generator (on:off 50:50 and 70:30) were explored in order not to waste unnecessary energy to reinforce the green aspect. Determining the acoustic power within fixed time using two pulse modes demonstrates the power difference in the experiments. In any case, the commercially available ultrasound generator used could not be run in full continuous mode, probably because of electronic protection not to irreversibly damage the piezo-ceramics. The calculated acoustic powers are listed in Table 1. Acoustic power in each condition was calculated using Eq. (1):

(1) $$Acousticpower\left(W\right)=m∙c_{p}∙\left(\frac{\Delta T}{\Delta t}\right)$$

where m is the mass of water (kg), c_p_ is the specific heat of water (4.18·10^3^ J/kg°C), ΔT is the temperature change (°C) and t is time (s).

**Table 1:** Determined acoustic powers using different ultrasound conditions and two different starting temperatures.

**Experimental conditions**	**Acoustic power 22 °C (W)**	**Acoustic power 150 °C (W)**
70:30 70 % with added pressure (3 bar)	91	46
50:50 70 % with added pressure (3 bar)	52	35
70:30 70 % with no added pressure	29	17
50:50 70 % with no added pressure	21	14

The obtained data show that the selected operating conditions for this present study have a major effect on the delivered acoustic power. Whereas added pressure increases the delivered acoustic power, a higher starting temperature leads to lower acoustic power values. When measurement was started at room temperature, a 70:30 pulse with added pressure delivered 4.4 times higher acoustic power compared to the 50:50 pulse without added pressure. A similar effect was observed when measurement started at 150 °C, although the difference was slightly less because of the negative impact of the increased temperature on the cavitational activity.

#### 2.2.2 Ultrasound-assisted hot water treatment

Ultrasound-assisted fractionation was performed using the setup described in section 2.2.1. In the experiments, 1.25, 2.5 or 5 g of sawdust and 110 mL of water were mixed. Experiments were conducted either without additional pressure or with additional pressure of 3 bar, applied with compressed air before heating. The reactor was heated in an oil bath to 150 ˚C before starting the ultrasound irradiation. Oil-bath preheating allowed a temperature of 180 ˚C to be reached during the ultrasound treatment, the necessary temperature to enable hemicellulose hydrolysis as emphasized above. When the temperature inside the reactor reached 150 ˚C, ultrasound was applied for 30 min on pulse mode (either 50:50 or 70:30) at 70 % of amplitude. After sonication, the system was allowed to cool down for 10 min in the oil bath and then moved to a cold water bath. The liquid and solid phases were separated using centrifugation (Biofuge stratos, 4500 rpm, 3 min). The solid fraction was washed with water under vacuum filtration using a glass fiber filter (Sartorius Stedim Biotech GmbH, 0.45 µm) and dried overnight in an oven at 55 ˚C. The solid yield was calculated using Eq. (2). The liquid phase was analyzed with HPLC, and the pH was measured. Experiments were conducted as duplicates and reference treatments were performed in the same reactor without ultrasound (i.e., silent mode), using magnetic stirring (500 rpm) and the temperature was controlled with an oil bath.

(2) $$\mathrm{Solidyield}-\%=\frac{m_{\mathrm{final}}}{m_{\mathrm{initial}}}*100\%,$$

where m_final_ is the solid fraction’s mass after the treatment and m_initial_ is the mass of the initial sawdust loaded in the reactor.

For simultaneous furfural production, 2.5 g of sawdust was mixed with 1 %, 2.5 % or 5 % (v/v) formic acid aqueous solution. Experiments were conducted with 70:30 pulse at 70 % amplitude and 3 bar of additional pressure for 30 min, since these parameters have been demonstrated to exhibit higher delivered acoustic power. Also, extended experiments were performed by maintaining the temperature at 180 ˚C (±2 °C) for another 30 min without ultrasound after 30 min ultrasound treatment. In extended experiments the treatment with 2.5 % formic acid was conducted as duplicate for estimating variance in furfural yield. Reference treatments were conducted without ultrasound. The furfural yield was calculated using Eq. (3) considering the C5 sugar content of the sawdust.

(3) $${\mathrm{Yield}-\%}_{\mathrm{furfural}}=\frac{c_{\mathrm{furfural}}}{\left(\frac{\frac{m_{\mathrm{sawdust}*0.298*0.7}}{132.12}*M_{\mathrm{furfural}}}{V}\right)}*100\%$$

Where c_furfural_ is the measured furfural concentration (g/L), 132.12 is the molar mass of anhydro xylose unit (g/mol) and m is the mass of sawdust (g), 0.298 describes the mass percentage of hemicellulose in birch sawdust, 0.7 describes the mass percentage of C5 sugars in birch hemicellulose and V is the volume of liquid (L).

The theoretical pH values of the formic acid solutions were calculated using Eq. (4):

(4) $$\mathrm{pH}=-log\sqrt{\frac{V*\rho }{M*V_{\mathrm{solution}}}*1.8*10^{-4}}$$

where V is the volume of added formic acid, ρ is the density of formic acid, M is the molar mass of formic acid, V_solution_ is the total volume of solution and 1.8*10^-4^ is the dissociation constant of formic acid at 25 °C.

### 2.3 Liquid chromatography analysis

After the post-treatment of both the ultrasonic and silent experiments, the furfural and HMF contents of the liquid fraction were quantified using HPLC equipped with a Perkin Elmer series 200 pump, UV/vis detector, and Atlantis dC18 column (5 μm, 4.6*150 mm). Isocratic elution was used with an eluent composition of water:methanol (90:10 v/v) with 0.1 % TFA and 0.9 mL/min flowrate. The injection volume was 5 µL. Furfural and HMF were detected at 277 nm. Each sample was measured twice, and the results were calculated as an average of two injections.

The glucose and xylose contents were analyzed with a Waters I-Class Plus UPLC system equipped with an ELS-detector and ACQUITY BEH Amide column (1.7 µm, 2.1*150 mm). The analyses were performed with the column heated to 45 ˚C, a flow rate of 0.4 mL/min, and an injection volume of 1 µL (two injections per sample). The detector parameters were 350 kPa using nitrogen gas, 50 ˚C, gain 6, and 8 min linear gradient elution 0 % (A) water with 0.2 % TEA and 100 % (B) ACN with 0.2 % TEA to 30 % (A) and 70 % (B).

### 2.4 Solid fraction analysis

X-ray diffraction (XRD) analysis was conducted using a PANalytical X́Pert Pro device (Malvern Panalytical, Almelo, the Netherlands) with CuKɑ1 radiation (λ = 1.54060 Å) at 45 kV and 40 mA. The measuring range of 2θ and step size were 6–90° and 0.0170°, respectively. The analysis was carried out at 25 ˚C. The crystallinity index (CrI) of analyzed samples was calculated using the method described by Segal et al. (Eq. (5) [42],

(5) $$\mathrm{CrI}=\frac{I002-I_{\mathrm{am}}}{I_{002}}*100\%$$

where I_002_ is the maximum intensity of the 002-lattice diffraction and I_am_ is the amorphous background. The variance between and within samples was evaluated by repeating measurements for solids obtained from treatment conducted with 2.5 g sawdust loading. The Mann-Whitney *U* test was used to evaluate the significance of CrI difference between ultrasound and silent treatments at significance level of 0.05.

The obtained solid fractions were imaged with a Sigma Zeiss field-emission scanning electron microscope device (FESEM, Carl Zeiss Microscopy GmbH, Jena, Germany) to evaluate the effect of the treatments on particle size and wood structure. The results related to particle size were supported by sieving samples through test sieves (840, 500, 250, 150 μm). The results were calculated as m%.

## 3 Results and discussion

### 3.1 Ultrasound-assisted fractionation

The design of an ultrasonic reactor capable of ultrasound irradiation while coping with monitoring/controlling high temperature and pressure was the first challenge. Due to the protection of the power supply from overloading by shutting the power module down, the pulse mode was used instead of the continuous mode. The pulse mode enabled sonication at higher temperatures and pressures without shutting down the power module. It was noted that adding pressure to the system was beneficial for carrying out the experiments at high temperature, whereas it was challenging to start sonication without additional pressure. When 3 bar was added to the system before treatment, the sonication started without challenge and seemed to be more stable with a “regular” sound. Without additional pressure, sonication was unstable when the temperature was close to 180 ˚C, with apparent dropouts in heard sound frequencies.

**Fig. 3:**
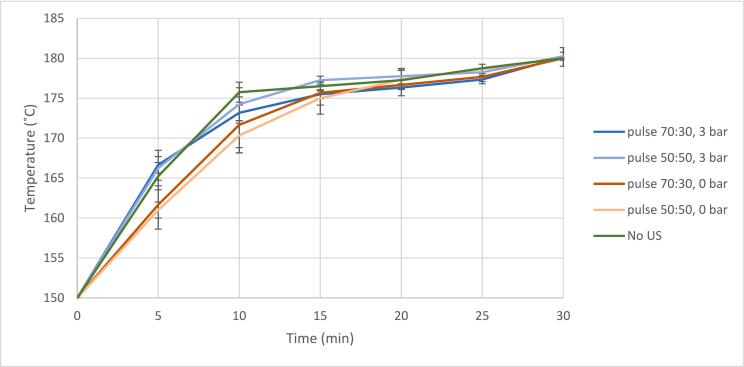
Heating profile of ultrasound experiments (lines) and standard deviation of temperature (°C; error bars).

Due to varying pressure and ultrasound parameters, the heating profile had to be stabilized to minimize the variation of results caused by the heating rate. Most of the variation in heating profile occurred at the beginning of the ultrasound treatment. However, a suitable heating profile with an acceptable level of deviation was achieved (Fig. 3). With and without an additional 3 bar pressure, the end pressure was 11 and 9 bar, respectively.

**Fig. 4:**
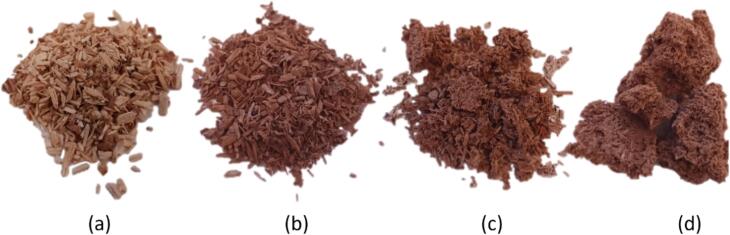
Effect of ultrasound on birch sawdust (2.5 g) fractionation in hot water (temperature increase from 150 °C to 180 °C over 30 min). (a) Birch sawdust, (b) in hot water treated sawdust (oil bath), (c) with ultrasound (70:30 pulse) in hot water treated sawdust, and (d) with ultrasound (70:30 pulse, 3 bar) in hot water treated sawdust.

No notable difference in solid yield was observed between ultrasound (with/without additional pressure) and corresponding silent treatments. A slight effect on solid yield was observed between different sawdust loadings. With 1.25, 2.5 and 5 g of sawdust, the solid yields were 61–64 %, 64–68 %, and 66–71 %, respectively indicating that lower solid to liquid ratio is slightly more efficient for dissolution of wood components, most likely hemicellulose. However, additional pressure influenced the physical appearance of sawdust (Fig. 4). When pressure of 3 bar was applied, obtained solids were dark brown, agglomerated, and finer (Fig. 4d) than without additional pressure (Fig. 4c). However, even without additional pressure, the structure of sawdust was clearly more disrupted compared to reference reactions without ultrasound. Ultrasound-treated sawdust was also fluffier compared to silent treatments. Without ultrasound, the sawdust structure was preserved regardless of the treatment conditions.

The liquid fractions were yellow to orange after all experiments. The harsher the experimental operating conditions were, the more intense (closer to orange) the color of the obtained liquid was. Also, with additional pressure (3 bar), the resulting liquid phase was clear while without added pressure the liquid fraction was turbid. Only a slight amount of furfural was produced (1–3 %, ±1 %) in all the experiments. The maximum furfural yield in all experiments was 2.9 %, while the HMF was not quantified due to a very low HPLC response. Borrega et al. obtained similar results at 180 °C, demonstrating that the applied temperature was not favorable for furfural or HMF production [23]. The pH of the liquid phase varied between 3 and 3.5, with and without ultrasound, which is in line with previous studies using similar temperatures for hot water extraction [23,[43], [44], [45]].

### 3.2 Formic acid-assisted furfural production

Formic acid was selected as the acid catalyst, since the acidic catalyzing agent should be weak enough not to damage the ultrasound probe, but also effective enough for hydrolysis and a conversion reaction. Also, environmental aspects as well as sustainability were considered [46,47]. For simultaneous furfural production, 1 %, 2.5 % and 5 % (v/v) formic acid aqueous solution was combined with ultrasound treatment, where 2.5 g of sawdust was mixed with 110 mL of liquid. Ultrasound was applied for 30 min with 70:30 pulse and 70 % amplitude. Additional pressure of 3 bar was added to the system, since the acoustic calorimetry measurement showed that with this additional pressure, cavitation effects were maximized. As previously described, the temperature was first raised to 150 ˚C with an oil bath and then ultrasonic irradiation was enabled, allowing the temperature to reach 180 ˚C. After treatment, the pH of the liquid phase was 2, 1.7, and 1.5 with 1 %, 2.5 %, and 5 % formic acid solution, respectively. The pH values of the liquid fractions indicate that part of the hemicellulose’s acetyl groups released and formed acetic acid, since the theoretical pH values for 1 %, 2.5 %, and 5 % formic acid solutions are 2.2, 2, and 1.8, respectively.

**Fig. 5:**
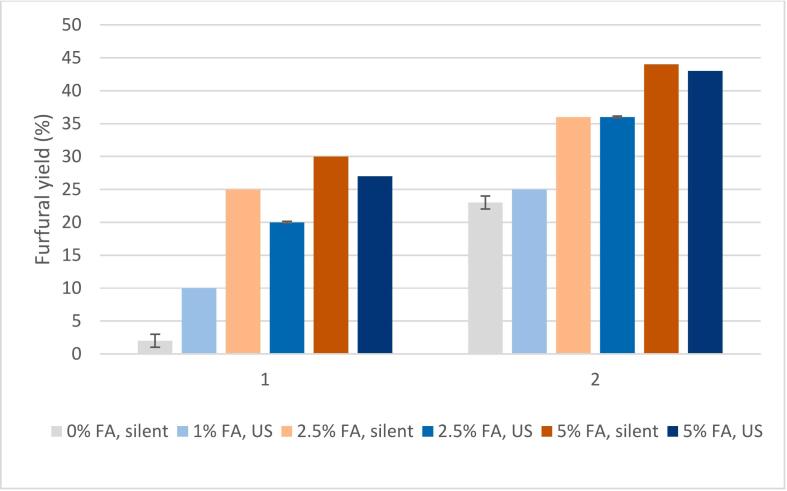
Effect of formic acid (FA) on the furfural yield in short (30 min) and extended (30 min treatment followed by 30 min hold at 180 °C) reactions using 2.5 g of sawdust, additional pressure of 3 bar, and 70:30 pulse at 70 % power in the ultrasound experiments.

Solid yields after formic acid treatments were 55–57 %, roughly 10 percentage points lower compared to experiments ran under neutral conditions which is most likely due to enhanced hemicellulose hydrolysis. Furfural yield increased with the increasing amount of acid (Fig. 5). When ultrasound treatment was conducted with 1 % formic acid, the furfural yield increased slightly, resulting in a 10 % yield. With 2.5 % and 5 % formic acid, the furfural yields were 20 % and 27 %, respectively, with ultrasound and 25 % and 30 %, respectively, without ultrasound.

Because it has previously been established that increasing the time increases furfural yield up to a certain point [48], extended experiments were conducted. After 30 min with ultrasound treatment, the temperature was kept at 180 ˚C for another 30 min with an oil bath without ultrasonic irradiation. For these experiments, 1 %; 2.5 % and 5 % (v/v) formic acid solutions were tested. The corresponding furfural yields were 25 %; 36 %, and 43 %, respectively (Fig. 5). Variation between duplicates conducted with 2.5 % acid was minor (0.15 %). The reference reaction (extended) without acid or ultrasound yielded 23 % of furfural, so it can be concluded that 1 % formic acid solution does not have a major impact on the conversion of C5 sugars into furfural. No notable difference in furfural yield in the presence of formic acid can be observed when comparing reactions with and without ultrasound, since the furfural yields with 1 %, 2.5 %, and 5 % formic acid in silent treatments were 29 %, 36 %, and 44 %, respectively. The formation of HMF was minor in all experiments. The maximum HMF concentration was 0.16 g/L, indicating that cellulose did not undergo major degradation during the treatment, which is understandable considering that cellulose undergoes hydrolysis at temperatures higher than 180–200 °C, ideally at 250–280 °C [22,38].

The effect of formic acid in similar conditions (without ultrasound) has previously been studied by Goldmann et al., who also noted that furfural production increases with increasing formic acid content (0–22 m%). It has been noted that increasing the temperature from 130 °C to 170 °C and the time from 1 h to 3 h enhance furfural production. However, with high formic acid concentrations, furfural yield decreased at 170 °C after 2  h. Furthermore, Goldmann et al. found that combined glucose and HMF yields remained relatively low at low temperatures and when using 0 or 7 m% formic acid, supporting the results achieved in the present study. The combined glucose and HMF yield was increased more significantly with higher formic acid concentrations at 170 °C [48]. Therefore, it can be concluded that a lower acid concentration is conducive to retaining cellulose in the solid fraction.

**Fig. 6:**
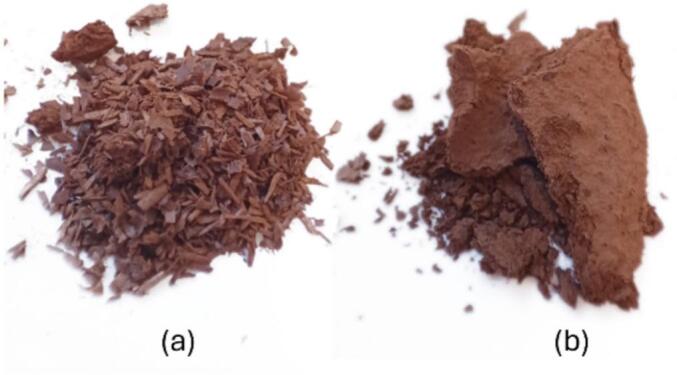
Birch sawdust treated with 2.5% formic acid in water (a) without ultrasound, and (b) with ultrasound.

Since the furfural yield was not affected by ultrasound, the most significant advantage of ultrasound was concluded to be related to the remaining solid phase rather than the conversion reaction in applied conditions. When acid was included in the ultrasound experiments, the solid material was even finer, almost powder-like and a little sticky, while without ultrasound, solids still had a sawdust-like structure despite the addition of acid (Fig. 6).

### 3.3 Sugar analysis

**Fig. 7:**
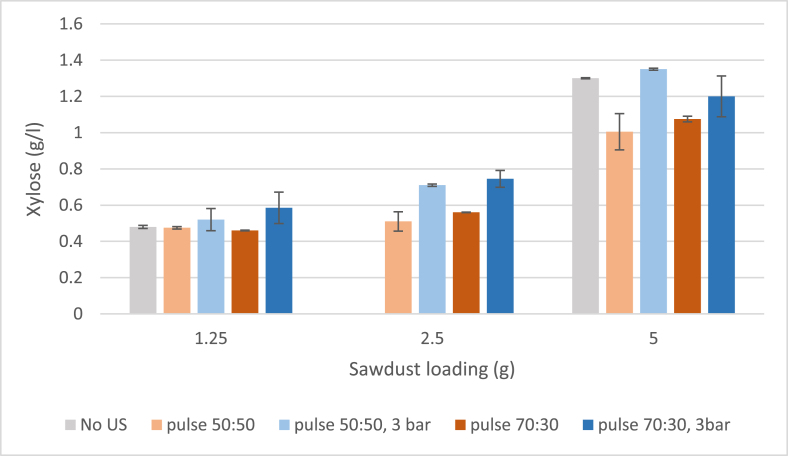
Xylose content in liquid fraction after ultrasound and with different sawdust loadings (g).

Similar to the effect of ultrasound on furfural yield, sugar analysis did not reveal any significant difference between the ultrasound and silent experiments. The xylose concentrations from hydrolysates obtained from silent treatments were measured for two hydrolysates obtained with 1.25 and 5 g sawdust loadings. However, a small difference was observed between ultrasound treatments when performed with and without additional pressure of 3 bar. Indeed, increasing the pressure was conducive to xylose yield compared to ultrasound treatments without additional pressure (Fig. 7). With added pressure, the sugar yields were comparable to silent treatments, whereas without added pressure, the sugar yields remained slightly lower compared to silent treatment, which yielded 1.3 g/L with 5 g sawdust loading. Sugar yields were also relatively small when considering the solid yield. Such low sugar concentrations detected by UPLC-ELSD are likely due to water soluble oligosaccharides.

A study by Kilpeläinen et al. found that birch treated with hot water at 180 °C yielded mostly oligosaccharides in the hydrolysate, even if most of hemicellulose was hydrolyzed into the liquid medium [37]. In the sugar analysis, only monosaccharides were detected, meaning that sugars with a higher degree of polymerization (DP) could not be quantified and their DP could not be detected. Also, the monosaccharide yield increased relatively to decreasing treatment of sawdust fraction, even when the difference in solid yield was relatively small; theoretical C5 sugar yields for 1.25, 2.5, and 5 g of sawdust were 2.7, 5.4, and 10.8 g/L, respectively.

**Fig. 8:**
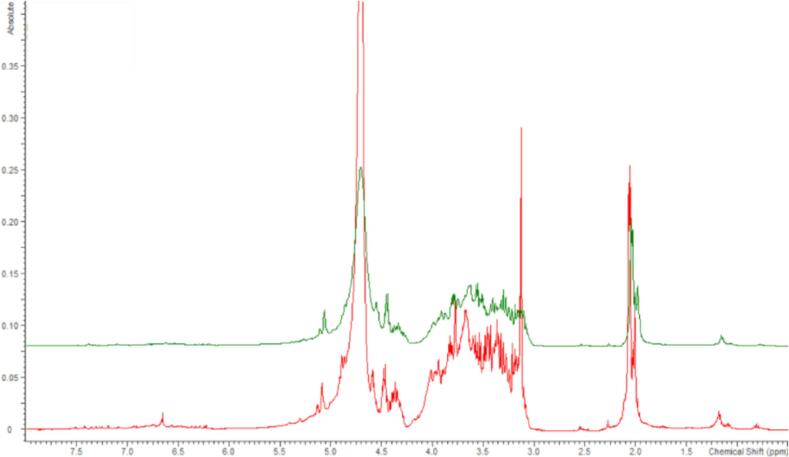
^1^H NMR spectra of solid residue of birch sawdust’s hydrolysate obtained from ultrasound treatment using 1.25 g sawdust, a 50:50 pulse, no added pressure (red), and 5 g sawdust, a 70:30 pulse, and added pressure of 3 bar (green).

Due to the incomplete detection of carbohydrates, two liquid fractions (from experiments conducted with 1.25 g sawdust, a 50:50 pulse, no added pressure, and 5 g sawdust, a 70:30 pulse, 3 bar added) were evaporated till dryness and mass balances of 90 % and 85 % were achieved, respectively. Mass balance was calculated by summing the solids from the treatment and evaporation to the monosaccharides detected with UPLC. The obtained solid residues were analyzed with ^1^H NMR using D_2_O as a solvent to support the earlier suggestions that the mass loss during the treatments was due to hemicellulose hydrolysis and that hemicellulose hydrolysis did not proceed completely to form only monosaccharides, which could be detected by UPLC-ELSD. The ^1^H NMR spectra were similar (Fig. 8) to the spectrum of birch xylan recorded previously by Teleman et al. [10] and showed strong signals at 3–4.1 ppm, where the xylan backbone protons are located [49], and signals at 4.25–4.7 ppm, which were previously assigned to anomeric H1 protons [10,49]. The signals in the region of 4.6–5.25 ppm overlapped with the D_2_O signal, but signals in that region were previously assigned to H2 and H3 of mono- and diacetylated xylan [10]. Other strong signals from the spectra were located at 2–2.2 ppm, which were assigned to acetyl groups found in birch hemicellulose [10,49]. Based on the obtained data and the literature, it can be concluded that the liquid fraction contained mainly water soluble hemicellulose, as suggested earlier.

**Fig. 9:**
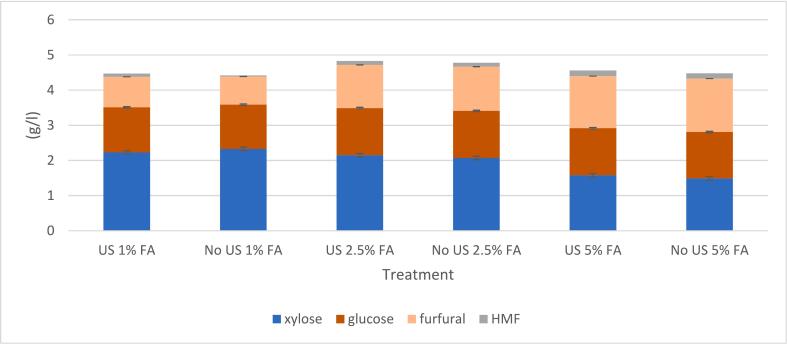
Liquid chromatography results of birch sawdust after hot water treatment with ultrasound (70:30 pulse, 30 min) and without ultrasound using a holding time of 30 min at the end of the treatment with an oil bath.

When experiments with formic acid were extended by 30 min, the total amount of detected compounds from treated sawdust in the liquid fraction remained almost constant, regardless of whether the experiments were performed with or without ultrasound. The highest amount of compounds detected from the hydrolysate was achieved when sawdust was treated with 2.5 % formic acid (Fig. 9), whereas the highest amount of furfural was obtained with 5 % formic acid and the highest amount of xylose with 1 % formic acid.

### 3.4 XRD analysis

The crystallinities (Crls) of obtained solid fractions were determined to evaluate the effect of ultrasound. It was noted that some parallel samples gave differing results for CrIs (variation < 1 %). The CrI was calculated as an average. Also, variation within samples (up to 1.4 %) was observed when samples were packed again and measured. Variation may be partly due to the heterogeneous nature of sawdust and irregular particle size (Table 2) and partly due to uneven exposure to ultrasound. The acoustic field is localized near the tip, so the intensity of ultrasound decreases both radially and axially when moving away from the tip, causing so-called dead zones in the system [16]. Despite the deviation, the lowest CrI values were obtained with 70:30 pulse and 3 bar additional pressure. The greatest difference between different conditions was observed with 2.5 g and 5 g sawdust loadings of which 2.5 g loading resulted in the lowest CrI (52 % ± 0.9 %) of experiments conducted in water. With 2.5 sawdust loading the treatments without additional pressure actually increased CrI compared to silent treatment. Obtained CrI values with 50:50 and 70:30 pulse without added pressure were 57 % (±0.9 %) and 58 % (±0.3 %), respectively and, 54 % (±0.4 %) with 50:50 pulse using added pressure. With 5 g loading, the CrI of sawdust, which was treated in silent conditions, was 58 %. The ultrasound treatments without added pressure using 5 g loading resulted in CrIs of 56–57 %. With added pressure (5 g loading) the CrIs of 54–56 % were obtained of which 70:30 pulse resulted in the lower value. Therefore, the results indicate that the added pressure could enhance the ultrasound effect especially with 70:30 pulse. Overall, CrI differences between samples that were treated in water were minor and the variation between and within samples complicates the interpretation of results. However, based on the results 70:30 pulse with added pressure was selected for the acid treatments since the lowest crystallinities were observed using those conditions.

**Table 2:** Sieving results (m-%) for sawdust, ultrasound treated sawdust and sawdust treated in silent conditions.

**Treatment**	**>840 μm**	**>500 μm**	**>250 μm**	**>150 μm**	**<150 μm**
−	16.3	67.4	95.5	99.5	0.5
Silent, 3 bar added	4.8	48.8	86.7	95.2	4.8
Ultrasound 70:30, 3 bar added	4.9	33.3	78.9	93.5	6.5
Ultrasound 70:30, no added pressure	14.7	64.1	92.3	97.4	2.6
Ultrasound 50:50, 3 bar added	14.8	57.7	91.9	98.7	1.3
Ultrasound, 70:30, 3 bar added, 2.5 % acid	6.7	22.7	57.1	76.5	23.5
Silent, 3 bar added, 2.5 % acid	10.6	60.6	92.4	99.2	0.8

**Fig. 10:**
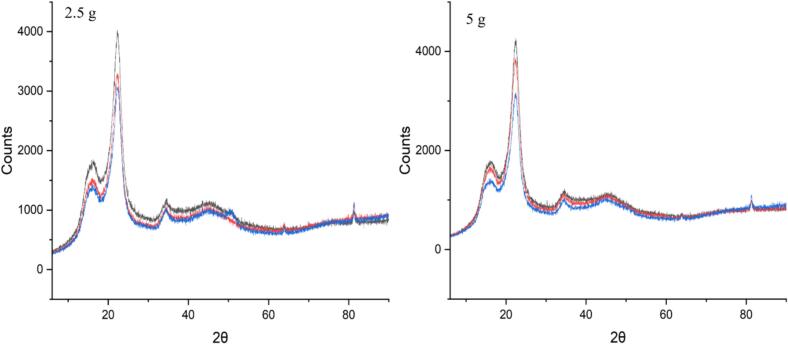
XRD diffractograms of sawdust treated in hot water under ultrasound for 30 min with a 70:30 pulse (red), a 70:30 pulse with additional pressure of 3 bar (blue), and without ultrasound for 30 min (black), increasing the temperature in all treatments from 150 °C to 180 °C.

**Fig. 11:**
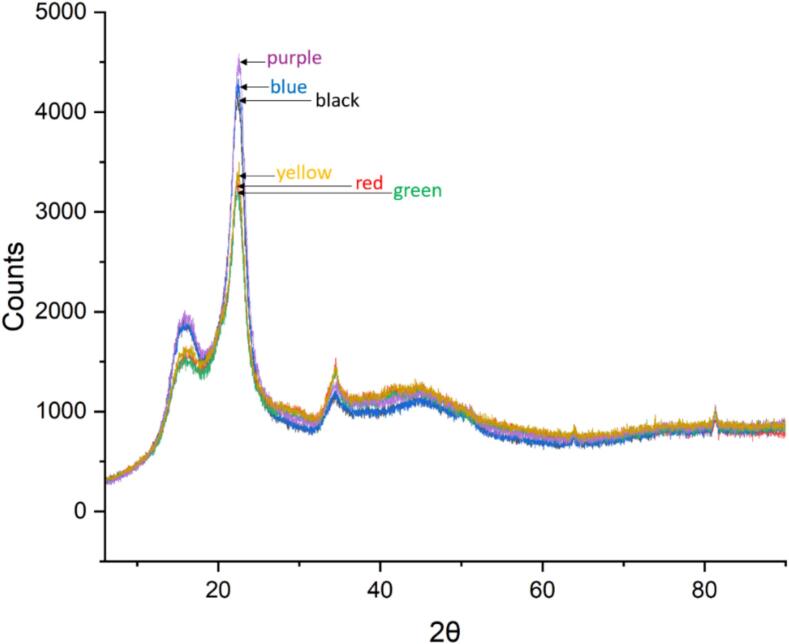
XRD diffractogram of sawdust treated with ultrasound (70:30 pulse, 3 bar, temperature raised from 150 °C to 180 °C over 30 min) in 1 % (red), 2.5 % (green), and 5 % (yellow) formic acid (v/v), and without ultrasound 1 % (black), 2.5 % (blue), and 5 % (purple) formic acid (v/v) and 30 min extended reaction time.

The diffractograms showed that ultrasound could decrease the crystal size slightly which could be concluded from peak widths. Fig. 10, Fig. 11 illustrates that the peaks of the ultrasound-treated samples are narrower than the silent treatments, especially when additional pressure was applied. Similar indications of ultrasound’s ability to decrease crystal size were previously observed by Sumari et al. [29].

When formic acid was added to the system, the effect of ultrasound was more noticeable. Compared to silent treatments, ultrasound reduced the crystallinity of solid material with all the used formic acid concentrations (Fig. 11). It should be noted that the solid yield was relatively constant, 55–57 %, despite the treatment method (ultrasound/silent) indicating equal removal of hemicellulose. Without ultrasound, the CrI remained relatively constant (55–56 %) despite the acid amount and the crystallinity was slightly higher compared to silent experiments conducted in water. That could be due to enhanced hydrolysis of amorphous hemicellulose in the presence of acid which can also be concluded from lower solid yield. When ultrasound was applied, CrI was 52 % with 1 % and 5 % acid and 50 % with 2.5 % acid. Therefore, it was concluded that silent experiments did not have similar effect on cellulose crystallinity compared to ultrasound. Also, the crystallinities of acid and ultrasound treated sawdusts were lower compared to those treated in water without ultrasound, despite lower solid yields in acid treatments. That is a clear indication that ultrasound assisted acid treatment decreased the crystallinity of cellulose. The greatest difference in CrI (6 percentage points/11 % change) between silent (CrI 56 %) and ultrasound experiments (CrI 50 %) was observed when 2.5 % formic acid was used (Fig. 11). For evaluating the significance between CrIs obtained from ultrasound and silent experiments, Mann-Whitney *U* test was used. According to the test, the difference between two groups (1: ultrasound, 2: silent) was statistically significant (U_statistic_ < U_critical_).

### 3.5 Effect of ultrasound on particle size and appearance of sawdust

**Fig. 12:**
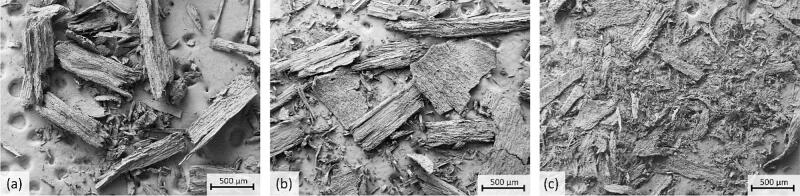
FESEM images (100x magnification) of (a) untreated sawdust, (b) sawdust (2.5 g) treated at 150–180 °C for 30 min without, and (c) with ultrasound (70:30 pulse, 3 bar).

From the FESEM images (Fig. 12), it was clear that ultrasound treatment could reduce the particle size more effectively compared to conventional heating with magnetic stirring. Also, based on sieving, untreated sawdust, sawdust treated in water using silent conditions and ultrasound contained 67 m%, 49 m% and 33–64 m% particles > 500 μm, respectively (Table 2). Furthermore, a 70:30 pulse with pressure of 3 bar was more effective for decreasing particle size compared to a 50:50 pulse or a 70:30 pulse without additional pressure. That could be concluded based on FESEM images and sieving, since the samples obtained from treatments conducted using 70:30 pulse with 3 bar added pressure, 50:50 pulse with 3 bar added pressure and 70:30 pulse without added pressure contained 33 m%, 56 m% and 64 m% particles > 500 μm, respectively. Therefore, it can be concluded that adding pressure to the system enhanced the ultrasound effect. It can also be seen from the images that ultrasound disrupted the wood structures more than the silent treatment (S1). The results support the conclusion made based on XRD that the most significant effect on sawdust can be obtained with 70:30 pulse with added pressure.

**Fig. 13:**
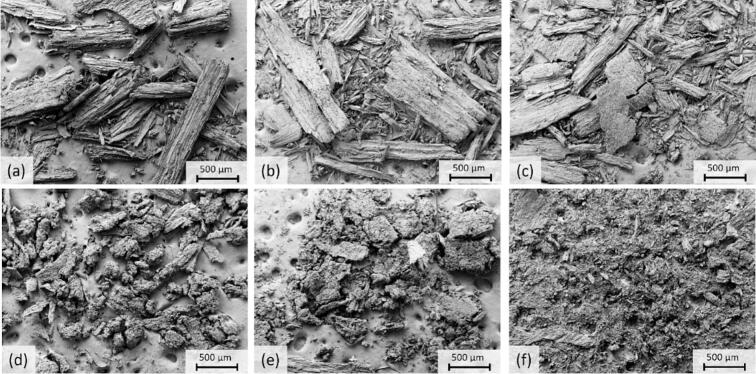
FESEM images (100x magnification) of sawdust (2.5 g) treated without ultrasound using (a) 1 %, (b) 2.5 %, and (c) 5 % formic acid and with ultrasound (70:30 pulse, 3 bar, 150–180 °C), using (d) 1 %, (e) 2.5 %, and (f) 5 % formic acid with 30 min extended reaction time in all treatments.

The FESEM images indicated that even 1 % formic acid clearly enhanced the ultrasound effect (Fig. 13), which was in line with the XRD (Fig. 11). Clear agglomeration of smaller particles was observed when ultrasound was applied together with formic acid. Wood structures could barely be observed and when increasing the acid content, the wood structures became even more difficult to distinguish in the images. Without ultrasound, particles remained sawdust-like, despite the addition of acid. More FESEM images are presented in S2. The conclusions made based on FESEM images were supported by sieving results which emphasized the synergistic effect of ultrasound and acid. The solid fraction from acid treatments (2.5 %) conducted using ultrasound contained 57 m% particles > 250 μm, while the corresponding value for solid obtained from silent treatment was 92 m%. Also, it was notable that solid from silent treatment contained only 1 m-% particles < 150 μm while corresponding value for ultrasound treated sawdust was 24 m%.

Sawdust treated in diluted acid with ultrasound was observed to differ greatly from sawdust treated without acid. It formed a sticky cake on the membrane when washing it during vacuum filtration. Based on both liquid and solid fraction analyses, the solid fraction contained lignin and carbohydrates, mostly cellulose. According to the literature, hydrolyzed wood and lignin-carbohydrate mixtures can be used for paper and plastic-like films, but the procedures tend to require long or harsh conditions [[50], [51], [52]]. Therefore, it would be worth studying in the future whether these kinds of films could be prepared with an ultrasound-assisted method, since the method used in this study combines two advantages, the short treatment time and the use of diluted weak acid.

## 4 Conclusions

Sawdust was ultrasonicated, at two pressures (ambient or 3 bar), without and in the presence of formic acid, raising the temperature from 150 °C to 180 °C over 30 min. The most effective conditions for disrupting the wood structure, decreasing the crystallinity and reducing particle size were ultrasound (70:30 pulse, added pressure of 3 bar), combined with formic acid. The ultrasound affected mainly the obtained solid fraction: the wood structure was totally disrupted, and the particle size was clearly reduced. The ultrasound treatment with 2.5 % formic acid resulted in significantly more (23 percentage points) fine particles (<150 μm) compared to silent experiment. It was demonstrated that the crystallinity of solid fraction could be decreased by up to 6 percentage points (11 % decrease) by ultrasound-assisted acid treatment, when compared to silent treatment, which enabled easier cellulose hydrolysis in further steps. Added pressure was observed to enhance the ultrasound effect related to solid fraction. However, no difference in liquid fraction was observed with used analytical methods. It would be interesting to study in the future if the ultrasound influences DP of soluble hemicellulose. Despite the lack of influence on liquid fraction, relatively high furfural yield (43–44 %) was obtained. Therefore, the treatment could be used simultaneously for furfural production, where higher acid content and longer time were favorable, and for pretreatment to utilize cellulose in further steps. Simultaneous conversion of hemicellulose and pretreatment of solid fraction facilitates the overall process even if based on obtained the results, ultrasound plays a key role only in pretreatment. These findings suggest great potential for conducting future complementary research for finding the optimal parameters for sawdust pretreatment, fractionation and conversion to platform chemicals. Solid fraction utilization for other purposes, for example, film, could also be studied in future.

## Author contributions

This research was conducted by Salla Kälkäjä. Formal analyses were performed by Salla Kälkäjä and Tao Hu, while the acoustic power measurements were conducted by Jean-Marc Lévêque. The first draft of the manuscript was authored by Salla Kälkäjä; and Katja Lappalainen, Jean-Marc Lévêque and Stéphane Baup commented on earlier versions. The application for research funding was undertaken by Katja Lappalainen, while the funding application for mobility between Finland and France was undertaken by Salla Kälkäjä. The work was supervised by Katja Lappalainen and Jean-Marc Lévêque. All authors read and approved the final manuscript.

## CRediT authorship contribution statement

**Salla Kälkäjä:** Conceptualization, Methodology, Formal analysis, Investigation, Writing – original draft, Writing – review & editing, Visualization, Funding acquisition. **Tao Hu:** Investigation, Writing – review & editing. **Stéphane Baup:** Resources, Writing – review & editing. **Jean-Marc Lévêque:** Conceptualization, Resources, Writing – review & editing, Supervision. **Katja Lappalainen:** Conceptualization, Writing – review & editing, Supervision, Project administration, Funding acquisition.

## Funding

The research was funded by the Kone Foundation (Grant Number 201903073). Finnish Forest Products Engineers and University of Oulu Graduate school enabled the mobility between University of Oulu and Université Grenoble Alpes.

## Declaration of competing interest

The authors declare that they have no known competing financial interests or personal relationships that could have appeared to influence the work reported in this paper.
