# Supplementary material for: The effect of ultrasound on birch sawdust during simultaneous pretreatment and hemicellulose’s chemical conversion
Source: Ultrason Sonochem. 2025 Mar 19;116:107318. doi: 10.1016/j.ultsonch.2025.107318 (PMC11981785; doi:10.1016/j.ultsonch.2025.107318)
Supplement: Supplementary Data 1 [file mmc1.docx]

| **FESEM Images of sawdust treated with and without ultrasound in water** | | | | |
| --- | --- | --- | --- | --- |
| **US 50:50 pulse** | **US 50:50 pulse, 3 bar** | **US 70:30 pulse** | **US 70:30 pulse, 3 bar** | **Silent** |
|  |  |  |  |  |
|  |  |  |  |  |
|  |  |  |  |  |
|  |  |  |  |  |
|  |  |  |  |  |
|  |  |  |  |  |
