# Supplementary material for: The effect of ultrasound on birch sawdust during simultaneous pretreatment and hemicellulose’s chemical conversion
Source: Ultrason Sonochem. 2025 Mar 19;116:107318. doi: 10.1016/j.ultsonch.2025.107318 (PMC11981785; doi:10.1016/j.ultsonch.2025.107318)
Supplement: Supplementary Data 2 [file mmc2.docx]

**FESEM Images of sawdust treated with ultrasound assisted formic acid treatment**

**1 % Formic acid**

| **With US (70:30, 3bar)** | **Silent** |
| --- | --- |
|  |  |
|  |  |
|  |  |
|  |  |
|  |  |
|  |  |

**2.5% FA**

| **With US (70:30, 3 bar)** | **Silent** |
| --- | --- |
|  |  |
|  |  |
|  |  |
|  |  |
|  |  |
|  |  |

**5% FA**

| **With US (70:30, 3 bar)** | **Silent** |
| --- | --- |
|  |  |
|  |  |
|  |  |
|  |  |
|  |  |
|  |  |
